# Saratoga, 1896

**Published:** 1896-09

**Authors:** 


### Editorial.

### SARATOGA, 1896.

THE convention period has come and passed into history. Whether this will mark an epoch is doubtful, nor was it expected. The true scientific and practical work of the dental profession, as stated in a recent article, must be sought for elsewhere, but that does not detract from the usefulness of these bodies, for their value cannot be found so much in the work accomplished as in the general binding influence they exercise over the widely-distributed circle of workers. Without a central delegated body there would be a want of uniformity or lack of completeness in professional life, and hence, whatever may be thought of these national conventions, there is no probability of their passing away in this generation. That the machinery of professional work could be improved there can be no question; but if this should ever be effected it will be accomplished by slow processes of evolution through a higher conception of means to ends.

Saratoga has been the place of many gatherings of the dental fraternity. It, perhaps, would be difficult to define why every four or five years this place should seem to have sufficient attractions to secure a majority of the votes of the members of the American Dental Association, but such has been the fact. The reason for this may be found in the quiet beauty and restfulness of the place, for it is this, notwithstanding the general idea is exactly the opposite. This celebrated resort is just now undergoing a transition from the era of gambling and horse-racing to another and, it is to be hoped, a better time when the public will seek it for more laudable purposes. The present condition, however, is not altogether satisfactory to the many who seek to glean a living from the restless travelling American life. They have flourished best under the old reign of wickedness, but the time will come when the truer life will assert itself and demonstrate, as has been done in other cases, that true prosperity can never be founded on a basis of evil.

The various national conventions,—the National Association of Dental Faculties, the National Association of Dental Examiners, the Dental Technic Association, as well as the parent body, the American Dental Association, had full representation.

There was nothing of very great importance accomplished in either of the two first named, nor was it anticipated. Legislation for colleges has reached a point where it is better to advance slowly. The feeling was, however, very evident that a marked advance in the near future would be required, as it was evident that, with the extension of the curriculum, four years would soon be necessary, as well as an increase in the length of the term. There is probably no one of the national organizations which concentrates so much of interest as this society, and naturally so, for it embodies in its membership some of the best of the intellectual forces in the dental profession of the United States. With such a body of thinkers, meetings could not fail to be full of interest, and the yearly discussion of educational problems furnishes a means of centralizing questions which, when distributed to distant colleges, must exercise an influence for growth not to be over-estimated. The "Faculties," as it is familiarly called, was a creation made amid the most discouraging conditions that ever prevailed in dental education in this country, and when the late Professor Winder suggested such an organization, it was regarded as an almost hopeless effort. The attempt was made, and now, in 1896, the organization dominates the dental education of this country, and will continue to do this just as long as wise counsels prevail. There has been no tendency heretofore to use this power but for good, and it is hoped that those in control will avoid the rocks upon which so many similar organizations have been wrecked.

The tendency to legislate for everything in the United States is a serious and growing evil, and the multiplication of laws governing this profession indicates that the time will come when we will have reason to regret the increase of statutes shackling the free action of professional work. Within reasonable limit this is well, but in every direction the boasted freedom of the people will exist only in name, unless the unwise appeals to law are held in check. The National Association of Dental Examiners has an advisory work to do in this direction, and if this be properly used, many of the clouds that obscure our professional horizon may be dispersed. What the work of this organization was at Saratoga we are not at this writing informed, as its sessions are not open to the public; but it is an annually growing power, and what is true of the National Association of Dental Faculties is equally applicable to this body.

The meeting of the American Dental Association was not as satisfactory as could have been desired. It was fairly well attended, perhaps more so than could have been expected under the depressed conditions of the country. The so-called scientific side of dentistry was well represented in several able papers, but the practical was almost wholly neglected. It is difficult to determine why this should have been, for the past year has not been, by any means, a barren one in the direction named; indeed, it has undoubtedly in many respects been an advance over several previous years. While a general apathy seemed to prevail in the branches of operative and mechanical dentistry, it is probably due to other causes than a lack of interest.

It seems to the observing and reflective mind that dental association methods have reached a condition that should cause thought, and suggest the question, "Have we not arrived at a period when a change would be profitable?" Large bodies necessarily move slowly; but it is quite evident that whether fast or slow, they must move, or their usefulness will come to an end. The American Dental Association is an active, energizing society, and has by no means reached the period of senility, which comes to associations as well as individuals sooner or later. Its power for good cannot well be over-estimated, but that will cease if allowed to drift into a state of lethargy.

There is an anomalous condition existing at present in these United States, the tendency of which is not for the best interests of the dental profession. It is true of organized bodies, as it is of all the relations of life, that divided councils tend to weakness. Two national bodies in dentistry is a positive error, and it has been felt for years that this should not be permitted to continue.

The American Dental Association and the Southern Dental Association will meet next year (1897) at Old Point Comfort, and it is to be hoped that mutual sacrifices will be made looking towards a union of these two organizations. The state of divided interests should not be allowed to continue. With union and new life infused in both Associations, dentistry would, we are satisfied, spring into newness of life. That this is a general desire must have been evident from the expressions at Saratoga, and it is felt that this meeting, though not as full of good things as many preceding ones, will outrank them all, in that it marks an epoch of good feeling and a desire to harmonize all conflicting interests and gather all into one national body. That this was the feeling was evident in voting for a place of meeting next year. The desire to meet with our

Southern brethren caused the majority vote for Old Point Comfort. There can be no question but that Denver would have been selected had it not been felt that this important question must be settled. In 1898 the united body should go West, and Denver seems a happy compromise between California, in the far West, and Old Point Comfort, Niagara Falls, and Saratoga in the far East. The interest taken by the people of Denver in efforts to secure the meeting there next year was not only remarkable but was very satisfactory and complimentary to this body of dental workers, and while it was felt necessary to disappoint them, there is no reason why the succeeding meeting should not be held there; indeed, it is essential to the well being of the national organization that this should be decided upon at the proper time.

The conventions of 1896 having completed their work, we may confidently anticipate that their influence will be a continually aggregating force for the higher development of the profession they aim to represent and in the interest of which they have been organized. Let, then, Saratoga, 1896, be the beginning of the closing years of the century, an ending that should be replete with activity and in an extended growth in all things essential to the well-being of dentistry.

